# Spurious Hyperphosphatemia in Two Children With End-Stage Renal Disease: A Case Report

**DOI:** 10.7759/cureus.55818

**Published:** 2024-03-08

**Authors:** Fayha Salah Ahmed, Mohamedi Begum, Mouza Abdulla AlSharhan

**Affiliations:** 1 Clinical Biochemistry, Dubai Hospital/ Dubai Academic Health Corporation, Dubai, ARE; 2 Pathology and Laboratory Medicine, Dubai Hospital/ Dubai Academic Health Corporation, Dubai, ARE

**Keywords:** esrd (end stage renal disease), hemodialysis, hd catheter, alteplase, heparin, spurious hyperphosphatemia

## Abstract

Spurious hyperphosphatemia, also known as pseudo-hyperphosphatemia, refers to artifactually elevated serum phosphate values that do not correspond to their actual systemic levels.

Vascular access poses a significant challenge for individuals undergoing hemodialysis (HD) due to chronic kidney disease, primarily attributed to the elevated incidence of complications, such as infections or thrombosis associated with catheter use. To mitigate clotting risk during the inter-dialysis intervals, in recent years, a strategy involving the application of concentrated heparin (recombinant tissue plasminogen activator (rt-PA) e.g. alteplase) has been used. These infusions have a very high content of phosphorus, and if it is not removed sufficiently before collecting blood samples through the central venous catheter, it can cause erroneously high phosphate levels.

Here we report two cases of pseudo-hyperphosphatemia caused by contaminated blood samples obtained from an HD catheter from the pediatric nephrology department. Absurd values found in routine samples led us to investigate the reason for these results. Further investigations carried out by the laboratory biochemistry department showed that all the analytical checks and proficiency testing performance were within acceptable limits.

We hypothesized that there was a pre-analytical error such as possible contamination with the high phosphate content of heparin and alteplase solution in the catheter, contributing to the increased phosphate levels in these samples.

## Introduction

Elevated phosphate levels, known as hyperphosphatemia, stand out as a significant and independent risk factor contributing to cardiovascular calcification, cardiovascular morbidity, and mortality among individuals undergoing dialysis [1-3]. Regular monitoring of serum phosphate levels is a standard practice in patients receiving maintenance dialysis therapy, with heightened levels typically attributed to factors such as dietary non-adherence or insufficient use of oral phosphate binders.

We present a case report on pseudohyperphosphatemia caused by contaminated blood samples obtained from a hemodialysis (HD) catheter in two children with end-stage renal disease (ESRD).

## Case presentation

Case 1

A 14-year-old female patient with ESRD secondary to nephronophthisis was on long-term HD therapy with a tunneled central venous catheter (HD catheter) for the past six months with alternate days sessions, each session lasting for four hours.

Her comorbid conditions included anemia (thalassemia alpha carrier), pityriasis alba, and Senior-Loken syndrome, which is a rare disorder characterized by the combination of two specific features: nephronophthisis and an eye condition known as Leber congenital amaurosis.

Her medications included sevelamer (one tablet of 800 mg total daily) and cholecalciferol (10,000 units once per week). During each HD session, 5,000 units of heparin sodium were administered intraluminally two times a day as well as an intravenous injection bolus of 700 units of heparin sodium.

The first spurious hyperphosphatemia incident happened on 24/11/2020 when a sample was received from the ward and the initial phosphate value was 27.09 mg/dL. The test was repeated on the same day with a new sample collection but from a different site with direct IV access; the result for the new sample was 2.8 mg/dL.

The same scenario was repeated after three months when we got a sample with a critically high phosphate concentration of 13.5 mg/dL. Again this was repeated on the same day with a new sample collection from the same HD catheter after informing the nurse to follow the standard procedure for collecting blood samples through the HD catheter; the result came as 3.1 mg/dl. Biochemical data for Case 1 is tabulated in Table 1 below.

**Table 1:** Biochemical Data for Case 1

Parameters		Result values	Reference values and units
Sodium		134	131 - 145 mmol/L
Potassium		3.9	3.2 - 5.4 mmol/L
Chloride		97	96 - 111 mmol/L
Bicarbonate (HCO3)		20.9	20 - 28 mmol/L
Urea		122 ↑	12 - 40 mg/dL
Anion gap		17 ↑	6 - 14 mmol/L
Creatinine, blood		6.5 ↑	0.5-0.9 mg/dL
Calcium		9.8	8.9 - 10.2 mg/dL
25 OH Vitamin D Total (D2+D3)		34.6	> 30 ng/mL
Parathyroid hormone		499 ↑	15 - 65 pg/ml
First incident of pseudohyperphosphatemia on 24/11/2020			
Phosphate, blood	Initial value	27.09 ↑	2.7 - 4.5 mg/dL
	Repeated value	2.8	
Second incident of pseudohyperphosphatemia, three months later			
Phosphate, blood	Initial value	13.5 ↑	2.7 - 4.5 mg/dL
	Repeated value	3.1	

Case 2 

An 18-month-old female patient was diagnosed with ESRD secondary to dysplastic kidneys, ectopic dilated kidney, and reflux nephropathy. She was on long-term HD therapy on alternate days with each session lasting for a period of four hours through a tunneled central venous catheter (HD catheter).

Her comorbid conditions included congenital anomalies of the cervix, vagina, and external female genitalia.

Her dialysis sessions started with a bolus intravenous injection of 160 units of heparin sodium and were followed by four hours of heparin sodium IV infusion at a rate of 0.03 mL/hr. At the end of the dialysis session, the catheter was occasionally locked with alteplase, 0.9 mg into the red port and 1.1 mg into the blue port to prevent clotting and thus ensure adequate blood flow for dialysis.

Regular pre-dialysis monthly follow-up labs on 29/11/2020 showed a high phosphate concentration of 20.8 mg/dL which did not match her clinical condition. The test was repeated again and the result was 2.2 mg/dL; this halted probable medical intervention to treat the hyperphosphatemia. Biochemical data for Case 1 is tabulated in Table 2 below.

**Table 2:** Biochemical Data of Case 2

Parameters		Result values on 29/11/2020	Reference values and units
Sodium		137	131 - 145 mmol/L
Potassium		3.2	3.2 - 5.4 mmol/L
Chloride		95	96 - 111 mmol/L
Bicarbonate (HCO3)		28.9	20 - 28 mmol/L
Urea		19	12 - 40 mg/dL
Anion gap		15 ↑	6 - 14 mmol/L
Creatinine, blood		3.4 ↑	0.44 - 0.65 mg/dL
Calcium		10.5	8.9 - 10.2 mg/dL
25 OH Vitamin D Total (D2+D3)		51.3	> 30 ng/mL
Parathyroid hormone		705 ↑	15 - 65 pg/ml
Phosphate, blood	Initial value	20.8↑	2.7 - 4.5 mg/dL
	Repeated value	2.2	2.7 - 4.5 mg/dL

## Discussion

The purpose of this case report is to highlight the differential diagnosis of hyperphosphatemia in HD patients.

Spurious hyperphosphatemia has been reported to occur in cases of hyperbilirubinemia, hemolysis, paraproteinemia, and hyperlipidemia. The possibility of interference with the assay has been excluded in these two patients [4-6].

In both of the above cases, we were puzzled by the very high and fluctuating concentration of phosphorus. There was no evidence of bleeding, hemolysis, or rhabdomyolysis and thus we suspected a pre-analytical error.

After querying the dialysis nurses about the practice of blood collection from the HD catheter, we came to know that they were aspirating and discarding 2-5ml of blood before actually collecting the blood sample for laboratory orders. The nurses were following the same procedure stated in the hospital policy for blood collection through an HD catheter. We attribute the heightened phosphate concentration in blood samples to potential contamination with heparinized solution.

According to the manufacturer’s package insert, heparinized saline, commonly used, is buffered with a phosphate solution containing approximately 105mg/dL of phosphate. In the case of HD patients with a central line, when investigating hyperphosphatemia, it is crucial to consider the use of heparin or tissue plasminogen activator, as these substances can lead to inaccurately elevated serum phosphate levels due to improper sampling [7]. The study by Ball et al. concludes that even minimal heparin contamination can result in a significant rise in serum phosphate levels [8]. Therefore, strict adherence to precise blood drawing procedures from indwelling catheters is imperative to prevent sample contamination, thus avoiding the generation of inaccurate laboratory values or reports. 

Clinicians, particularly nephrologists, should remain vigilant regarding the potential of contamination of blood samples with phosphate, a consequence of inadequate rinsing of HD catheters when employing phosphate-containing anticoagulants such as heparin and recombinant tissue plasminogen activators (rt-PA) [9].

The Clinical and Laboratory Standards Institute (CLSI) advises that when drawing blood from an indwelling catheter or vascular access device (VAD), a protocol should be followed. Specifically, the line should be flushed with 5mL of saline, and subsequently, the first 5 mL of blood or the equivalent of six dead-space volumes of the VAD should be discarded before collecting any blood sample [10]. Taking into consideration our findings and applying this guidance to patients with end-stage renal disease having a tunneled catheter, we recommend the routine practice of drawing and discarding 5-6 mL of blood from each port. This precautionary measure aims to prevent interference with residual drug content in the line. It is essential to emphasize strict adherence to this procedure among all care providers. As a result of our study, the hospital policy has been updated to incorporate these standard procedures for sample collection from vascular access devices into practical application. 

**Figure 1:**
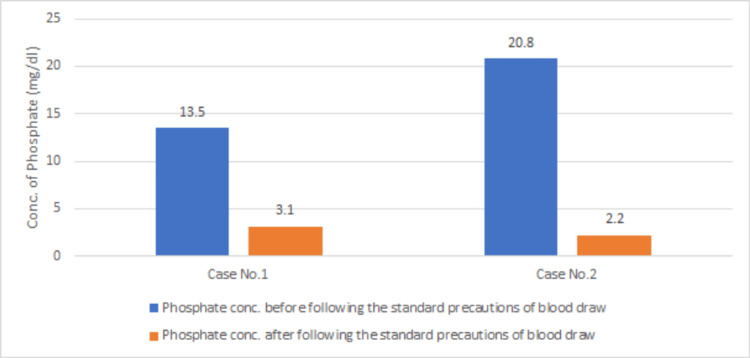
Comparison of phosphate concentration (mg/dL) before and after following the standard precautions in Cases 1 and 2 conc.: concentration

A comparative analysis of phosphorus measurements in both initial and repeated samples, following standard precautions, revealed notable variations in phosphorus concentration, with significant fluctuations from one sample to another (Figure 1).

## Conclusions

The concept of pseudohyperphosphatemia resulting from heparin flush and its contribution to preanalytical errors often goes unnoticed. Hence, it is pertinent to include the consideration of spurious hyperphosphatemia in the differential diagnosis when encountering unexplained elevated phosphorus levels with normal serum calcium in patients undergoing HD. Nephrologists should remain cognizant of the potential contamination of blood samples with phosphate, a consequence of inadequate rinsing of HD catheters, particularly when employing phosphate-containing anticoagulants like heparin and rt-PA. This awareness is crucial for accurate diagnostic interpretation and appropriate clinical management.

In conclusion, there was a high risk of malpractice in such cases and we emphasize the critical importance of adhering to stringent blood draw procedures from HD catheters. This practice is essential to prevent sample contamination, which could lead to inaccurate laboratory values and subsequently impact the clinical management of patients. Maintaining a high standard in blood collection protocols is paramount for ensuring the reliability and validity of laboratory results, thus facilitating more accurate and effective patient care.
